# (*Z*)-3-*o*-Tolyl-3-(*p*-tol­yloxy)acrylonitrile

**DOI:** 10.1107/S1600536812025858

**Published:** 2012-06-13

**Authors:** Li Zhou, Chuan-Hu Wang, Mi Zhou

**Affiliations:** a Department of Applied Chemistry and Environmental Engineering, Bengbu College, Bengbu 233030, Anhui, People’s Republic of China

## Abstract

The title compound, C_17_H_15_NO, exists in a *Z* conformation. The dihedral angle between the O-bonded benzene ring and the vinyl plane is 80.97 (18)° while the dihedral angle between the rings is 80.06 (10)°. In the crystal structure, no classical hydrogen bonds occur.

## Related literature

For general background to acrylonitrile compounds and their biological, medical and pharmacological properties, see: Boedec *et al.* (2008 ▶); Napolitano *et al.* (2001 ▶); Reggio *et al.* (1998 ▶).
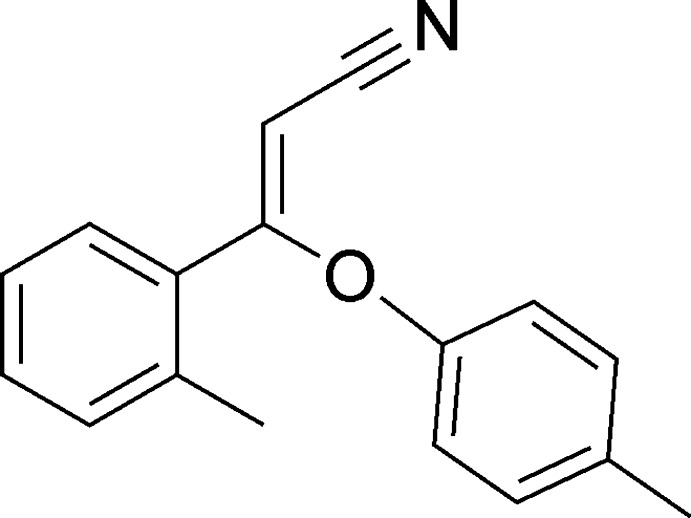


## Experimental

### 

#### Crystal data

C_17_H_15_NO
*M* *_r_* = 249.30
Tetragonal, 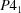

*a* = 9.8731 (6) Å
*c* = 14.2455 (17) Å
*V* = 1388.6 (2) Å^3^
*Z* = 4
Mo *K*α radiation
μ = 0.07 mm^−1^
*T* = 296 K
0.40 × 0.37 × 0.35 mm

#### Data collection

Bruker APEXII CCD diffractometer
Absorption correction: multi-scan (*SADABS*; Bruker, 2004 ▶) *T*_min_ = 0.971, *T*_max_ = 0.975
2539 measured reflections
1277 independent reflections
1028 reflections with *I* > 2σ(*I*)
*R*_int_ = 0.028

#### Refinement

*R*[*F* ^2^ > 2σ(*F* ^2^)] = 0.041
*wR*(*F* ^2^) = 0.111
*S* = 1.03
1277 reflections
172 parameters
1 restraint
H-atom parameters constrained
Δρ_max_ = 0.11 e Å^−3^
Δρ_min_ = −0.16 e Å^−3^

### 

Data collection: *APEX2* (Bruker, 2004 ▶); cell refinement: *SAINT* (Bruker, 2004 ▶); data reduction: *SAINT*; program(s) used to solve structure: *SHELXS97* (Sheldrick, 2008 ▶); program(s) used to refine structure: *SHELXL97* (Sheldrick, 2008 ▶); molecular graphics: *SHELXTL* (Sheldrick, 2008 ▶); software used to prepare material for publication: *SHELXTL* and *PLATON* (Spek, 2009 ▶).

## Supplementary Material

Crystal structure: contains datablock(s) I, global. DOI: 10.1107/S1600536812025858/rk2361sup1.cif

Structure factors: contains datablock(s) I. DOI: 10.1107/S1600536812025858/rk2361Isup2.hkl

Supplementary material file. DOI: 10.1107/S1600536812025858/rk2361Isup3.cml

Additional supplementary materials:  crystallographic information; 3D view; checkCIF report

## Figures and Tables

**Fig. 1.**
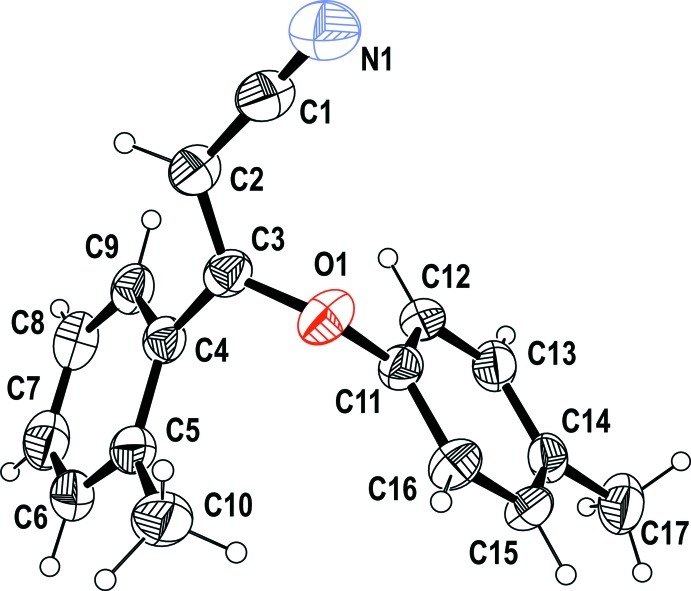
The molecular structure of the title compound, showing the atom-numbering scheme. Displacement ellipsoids are drawn at the 30% probability level. H atoms are presented as a small spheres of arbitrary radius.

## supplementary crystallographic information

### Comment

Acrylonitrile compounds have been widely found in dyes, agrochemicals, pharmaceutically active compounds, materials and natural products. Recent studies indicate that acrylonitrile compounds have broad range of biological, medical and pharmacological properties (Boedec *et al.*, 2008; Napolitano *et al.*, 2001; Reggio *et al.*, 1998).

As part of our interest in these materials, we report here the crystal structure of the title compound C_17_H_15_NO. The molecular structure of the title compound is shown in Fig. 1. The dihedral angle between phenol ring and vinyl plane is 80.97 (18)°. The any hydrogen bonds in crystal structure are not found.

### Experimental

For the preparation of the title compound, under nitrogen atmosphere, a sealable reaction tube equipped with a magnetic stirrer bar was charged with (*Z*)-1-(2-bromo-1-(*p*-tolyloxy)vinyl)-2-methylbenzene (1.0 mmol), K_4_Fe(CN)_6_ (0.20 mmol), Pd(O*Ac*)2 (0.01 mmol), P*Ph*_3_ (0.02 mmol) and *DMF* (2.0 ml). Then the reaction vessel placed in an oil bath at 393 K for 12 h and it was cooled to room temperature and diluted with ethyl acetate, washed with brine, dried with MgSO_4_. After the solvent was removed under reduced pressure, the residue was purified by column chromatography on silica gel to afford the product. Single crystals suitable for X-ray diffraction were prepared by slow evaporation of a solution of the title compound in dichloromethane at room temperature for three days.

### Refinement

All H atoms attached to C atoms were fixed geometrically and treated as riding with C—H = 0.93Å (for aryl H) and *U*_iso_(H) = 1.2*U*_eq_(C); C—H = 0.96Å (for methyl H) and *U*_iso_(H) = 1.5*U*_eq_(C) .

### Crystal data

**Table:**

C_17_H_15_NO	*D*_x_ = 1.192 Mg m^−^^3^
*M**_r_* = 249.30	Mo *K*α radiation, λ = 0.71073 Å
Tetragonal, *P*4_1_	Cell parameters from 2539 reflections
Hall symbol: P 4w	θ = 2.9–25.0°
*a* = 9.8731 (6) Å	µ = 0.07 mm^−^^1^
*c* = 14.2455 (17) Å	*T* = 296 K
*V* = 1388.6 (2) Å^3^	Block, colourless
*Z* = 4	0.40 × 0.37 × 0.35 mm
*F*(000) = 528	

### Data collection

**Table:**

Bruker APEXII CCD diffractometer	1277 independent reflections
Radiation source: fine-focus sealed tube	1028 reflections with *I* > 2σ(*I*)
Graphite monochromator	*R*_int_ = 0.028
ω–scans	θ_max_ = 25.0°, θ_min_ = 2.9°
Absorption correction: multi-scan (*SADABS*; Bruker, 2004)	*h* = −8→10
*T*_min_ = 0.971, *T*_max_ = 0.975	*k* = −1→11
2539 measured reflections	*l* = −16→9

### Refinement

**Table:**

Refinement on *F*^2^	Primary atom site location: structure-invariant direct methods
Least-squares matrix: full	Secondary atom site location: difference Fourier map
*R*[*F*^2^ > 2σ(*F*^2^)] = 0.041	Hydrogen site location: inferred from neighbouring sites
*wR*(*F*^2^) = 0.111	H-atom parameters constrained
*S* = 1.03	*w* = 1/[σ^2^(*F*_o_^2^) + (0.0658*P*)^2^ + 0.0136*P*] where *P* = (*F*_o_^2^ + 2*F*_c_^2^)/3
1277 reflections	(Δ/σ)_max_ < 0.001
172 parameters	Δρ_max_ = 0.11 e Å^−^^3^
1 restraint	Δρ_min_ = −0.16 e Å^−^^3^

### Special details

**Table:**

Geometry. All s.u.'s (except the s.u. in the dihedral angle between two l.s. planes) are estimated using the full covariance matrix. The cell s.u.'s are taken into account individually in the estimation of s.u.'s in distances, angles and torsion angles; correlations between s.u.'s in cell parameters are only used when they are defined by crystal symmetry. An approximate (isotropic) treatment of cell s.u.'s is used for estimating s.u.'s involving l.s. planes.
Refinement. Refinement of *F*^2^ against ALL reflections. The weighted *R*-factor *wR* and goodness of fit *S* are based on *F*^2^, conventional *R*-factors *R* are based on *F*, with *F* set to zero for negative *F*^2^. The threshold expression of *F*^2^ > σ(*F*^2^) is used only for calculating *R*-factors(gt) *etc*. and is not relevant to the choice of reflections for refinement. *R*-factors based on *F*^2^ are statistically about twice as large as those based on *F*, and *R*-factors based on ALL data will be even larger.

### Fractional atomic coordinates and isotropic or equivalent isotropic displacement parameters (Å^2^)

**Table:**

	*x*	*y*	*z*	*U*_iso_*/*U*_eq_	
N1	0.6863 (4)	0.6337 (4)	−0.0783 (3)	0.1013 (11)	
O1	0.8869 (2)	0.4406 (2)	0.07086 (17)	0.0694 (6)	
C1	0.6991 (4)	0.6132 (4)	0.0003 (3)	0.0744 (10)	
C2	0.7108 (3)	0.5867 (3)	0.0981 (2)	0.0684 (9)	
H2	0.6554	0.6339	0.1395	0.082*	
C3	0.7979 (3)	0.4971 (3)	0.1321 (2)	0.0566 (8)	
C4	0.8057 (3)	0.4605 (3)	0.2332 (2)	0.0548 (7)	
C5	0.9274 (3)	0.4663 (3)	0.2842 (2)	0.0631 (9)	
C6	0.9215 (4)	0.4387 (4)	0.3795 (3)	0.0772 (10)	
H6	1.0008	0.4433	0.4146	0.093*	
C7	0.8028 (5)	0.4049 (4)	0.4239 (3)	0.0850 (11)	
H7	0.8028	0.3863	0.4879	0.102*	
C8	0.6850 (4)	0.3986 (4)	0.3743 (3)	0.0751 (10)	
H8	0.6046	0.3749	0.4041	0.090*	
C9	0.6856 (3)	0.4278 (3)	0.2792 (2)	0.0624 (8)	
H9	0.6048	0.4256	0.2457	0.075*	
C10	1.0598 (4)	0.5058 (4)	0.2403 (3)	0.0883 (12)	
H10A	1.1021	0.4271	0.2136	0.132*	
H10B	1.1180	0.5441	0.2872	0.132*	
H10C	1.0439	0.5714	0.1918	0.132*	
C11	0.9107 (3)	0.3006 (3)	0.0749 (2)	0.0553 (7)	
C12	0.8072 (3)	0.2094 (3)	0.0842 (2)	0.0616 (8)	
H12	0.7185	0.2389	0.0922	0.074*	
C13	0.8366 (3)	0.0734 (3)	0.0815 (2)	0.0684 (9)	
H13	0.7662	0.0115	0.0878	0.082*	
C14	0.9668 (3)	0.0254 (3)	0.0699 (2)	0.0619 (8)	
C15	1.0682 (3)	0.1212 (4)	0.0605 (2)	0.0664 (9)	
H15	1.1571	0.0923	0.0527	0.080*	
C16	1.0413 (3)	0.2578 (3)	0.0624 (2)	0.0665 (8)	
H16	1.1110	0.3203	0.0554	0.080*	
C17	0.9986 (5)	−0.1231 (4)	0.0674 (3)	0.0916 (12)	
H17A	0.9627	−0.1619	0.0108	0.137*	
H17B	0.9583	−0.1667	0.1207	0.137*	
H17C	1.0950	−0.1357	0.0689	0.137*	

### Atomic displacement parameters (Å^2^)

**Table:**

	*U*^11^	*U*^22^	*U*^33^	*U*^12^	*U*^13^	*U*^23^
N1	0.103 (3)	0.108 (3)	0.094 (3)	0.0227 (19)	−0.015 (2)	0.010 (2)
O1	0.0669 (13)	0.0668 (13)	0.0746 (14)	0.0157 (10)	0.0185 (11)	0.0127 (12)
C1	0.067 (2)	0.072 (2)	0.084 (3)	0.0144 (17)	−0.008 (2)	0.001 (2)
C2	0.063 (2)	0.064 (2)	0.078 (2)	0.0093 (15)	−0.0049 (17)	−0.0066 (17)
C3	0.0467 (16)	0.0494 (17)	0.074 (2)	−0.0008 (12)	0.0016 (15)	−0.0041 (15)
C4	0.0553 (18)	0.0434 (15)	0.0658 (18)	−0.0004 (12)	−0.0002 (15)	−0.0072 (14)
C5	0.061 (2)	0.0514 (17)	0.077 (2)	0.0031 (13)	−0.0074 (16)	−0.0109 (16)
C6	0.088 (3)	0.067 (2)	0.077 (2)	0.0161 (17)	−0.017 (2)	−0.0199 (19)
C7	0.121 (4)	0.067 (2)	0.068 (2)	0.018 (2)	0.005 (2)	−0.0021 (19)
C8	0.089 (3)	0.059 (2)	0.078 (2)	−0.0003 (17)	0.023 (2)	−0.0041 (18)
C9	0.0614 (19)	0.0491 (17)	0.077 (2)	−0.0019 (13)	0.0068 (17)	−0.0099 (15)
C10	0.057 (2)	0.091 (3)	0.117 (3)	−0.0069 (18)	−0.005 (2)	−0.017 (2)
C11	0.0558 (17)	0.0632 (18)	0.0469 (15)	0.0122 (13)	0.0033 (14)	−0.0001 (15)
C12	0.0468 (17)	0.076 (2)	0.0620 (19)	0.0082 (14)	−0.0024 (15)	−0.0101 (17)
C13	0.065 (2)	0.073 (2)	0.067 (2)	−0.0043 (15)	0.0040 (17)	−0.0162 (17)
C14	0.074 (2)	0.0666 (19)	0.0447 (14)	0.0082 (15)	0.0018 (16)	−0.0084 (15)
C15	0.0545 (18)	0.077 (2)	0.068 (2)	0.0224 (15)	0.0023 (16)	−0.0042 (18)
C16	0.0511 (17)	0.073 (2)	0.076 (2)	0.0045 (15)	0.0126 (16)	0.0005 (18)
C17	0.120 (3)	0.076 (2)	0.078 (2)	0.021 (2)	0.006 (2)	−0.013 (2)

### Geometric parameters (Å, º)

**Table:**

N1—C1	1.145 (5)	C10—H10A	0.9600
O1—C3	1.358 (4)	C10—H10B	0.9600
O1—C11	1.403 (4)	C10—H10C	0.9600
C1—C2	1.421 (5)	C11—C12	1.369 (5)
C2—C3	1.326 (4)	C11—C16	1.368 (4)
C2—H2	0.9300	C12—C13	1.374 (5)
C3—C4	1.487 (4)	C12—H12	0.9300
C4—C9	1.393 (4)	C13—C14	1.380 (5)
C4—C5	1.406 (4)	C13—H13	0.9300
C5—C6	1.386 (5)	C14—C15	1.383 (5)
C5—C10	1.501 (5)	C14—C17	1.500 (5)
C6—C7	1.373 (6)	C15—C16	1.375 (5)
C6—H6	0.9300	C15—H15	0.9300
C7—C8	1.362 (6)	C16—H16	0.9300
C7—H7	0.9300	C17—H17A	0.9600
C8—C9	1.384 (5)	C17—H17B	0.9600
C8—H8	0.9300	C17—H17C	0.9600
C9—H9	0.9300		
			
C3—O1—C11	119.2 (2)	H10A—C10—H10B	109.5
N1—C1—C2	178.3 (4)	C5—C10—H10C	109.5
C3—C2—C1	122.2 (3)	H10A—C10—H10C	109.5
C3—C2—H2	118.9	H10B—C10—H10C	109.5
C1—C2—H2	118.9	C12—C11—C16	120.8 (3)
C2—C3—O1	117.3 (3)	C12—C11—O1	121.8 (2)
C2—C3—C4	123.4 (3)	C16—C11—O1	117.2 (3)
O1—C3—C4	119.3 (3)	C11—C12—C13	118.9 (3)
C9—C4—C5	119.6 (3)	C11—C12—H12	120.6
C9—C4—C3	117.9 (3)	C13—C12—H12	120.6
C5—C4—C3	122.3 (3)	C12—C13—C14	122.4 (3)
C6—C5—C4	117.5 (3)	C12—C13—H13	118.8
C6—C5—C10	119.8 (3)	C14—C13—H13	118.8
C4—C5—C10	122.6 (3)	C13—C14—C15	116.8 (3)
C7—C6—C5	122.4 (4)	C13—C14—C17	122.2 (3)
C7—C6—H6	118.8	C15—C14—C17	121.0 (3)
C5—C6—H6	118.8	C16—C15—C14	122.0 (3)
C8—C7—C6	120.0 (4)	C16—C15—H15	119.0
C8—C7—H7	120.0	C14—C15—H15	119.0
C6—C7—H7	120.0	C11—C16—C15	119.2 (3)
C7—C8—C9	119.6 (4)	C11—C16—H16	120.4
C7—C8—H8	120.2	C15—C16—H16	120.4
C9—C8—H8	120.2	C14—C17—H17A	109.5
C8—C9—C4	120.8 (3)	C14—C17—H17B	109.5
C8—C9—H9	119.6	H17A—C17—H17B	109.5
C4—C9—H9	119.6	C14—C17—H17C	109.5
C5—C10—H10A	109.5	H17A—C17—H17C	109.5
C5—C10—H10B	109.5	H17B—C17—H17C	109.5
			
C1—C2—C3—O1	6.7 (5)	C7—C8—C9—C4	1.5 (5)
C1—C2—C3—C4	−175.6 (3)	C5—C4—C9—C8	−1.2 (4)
C11—O1—C3—C2	−135.9 (3)	C3—C4—C9—C8	−177.1 (3)
C11—O1—C3—C4	46.3 (4)	C3—O1—C11—C12	44.3 (4)
C2—C3—C4—C9	49.1 (4)	C3—O1—C11—C16	−140.2 (3)
O1—C3—C4—C9	−133.3 (3)	C16—C11—C12—C13	0.5 (5)
C2—C3—C4—C5	−126.8 (3)	O1—C11—C12—C13	175.8 (3)
O1—C3—C4—C5	50.8 (4)	C11—C12—C13—C14	0.1 (5)
C9—C4—C5—C6	0.0 (4)	C12—C13—C14—C15	−0.2 (5)
C3—C4—C5—C6	175.7 (3)	C12—C13—C14—C17	179.8 (3)
C9—C4—C5—C10	−177.6 (3)	C13—C14—C15—C16	−0.2 (5)
C3—C4—C5—C10	−1.9 (4)	C17—C14—C15—C16	179.9 (3)
C4—C5—C6—C7	0.9 (5)	C12—C11—C16—C15	−0.8 (5)
C10—C5—C6—C7	178.6 (3)	O1—C11—C16—C15	−176.4 (3)
C5—C6—C7—C8	−0.6 (5)	C14—C15—C16—C11	0.7 (5)
C6—C7—C8—C9	−0.7 (5)		

## References

[bb1] Boedec, A., Sicard, H., Dessolin, J., Herbette, G., Ingoure, S., Raymond, C., Belmant, C. & Kraus, J. L. (2008). *J. Med. Chem.* **51**, 1747–1754. 10.1021/jm701101g 18303828

[bb2] Bruker (2004). *APEX2*, *SAINT* and *SADABS* Bruker AXS Inc., Madison, Wisconsin, USA.

[bb3] Napolitano, A., Bruno, I., Rovero, P., Lucas, R., Peris, M. P. & Riccio, R. (2001). *Tetrahedron*, **57**, 6249–6255.

[bb4] Reggio, P. H., Basu, S., Barnett, J., Castro, M. T., Hurst, D. P., Seltzman, H. H., Roche, M. J., Gilliam, A. F., Thomas, B. F. & Stevenson, L. A. (1998). *J. Med. Chem.* **41**, 5177–5187. 10.1021/jm9801197 9857088

[bb5] Sheldrick, G. M. (2008). *Acta Cryst.* A**64**, 112–122. 10.1107/S0108767307043930 18156677

[bb6] Spek, A. L. (2009). *Acta Cryst.* D**65**, 148–155. 10.1107/S090744490804362X PMC2631630 19171970

